# Baseline observation of performance and inter-professional utilisation of institutional hospital electronic technologies to access and communicate key clinical information in a central london teaching hospital critical care unit

**DOI:** 10.1186/2197-425X-3-S1-A864

**Published:** 2015-10-01

**Authors:** R Chevrier, K Child, S Shah, T Best, P Hopkins

**Affiliations:** King's College Hospital, King's Critical Care Units, London, United Kingdom

## Introduction

Optimisation of communication in critical care is known to improve quality including patient outcomes [1]. Electronic systems have been shown to facilitate improved access to information and its transfer in this setting, but effectiveness is dependent on design [2, 3].

## Objectives

Prior to implementation of a bespoke clinical information system in one of the largest critical care units in the UK, we conducted a baseline assessment of the utilisation and performance of the existing hospital electronic medical record (EMR) within critical care.

## Methods

Local research ethics approval was obtained. We measured electronic system speed and accessibility (pathology results; imaging archive; PDF documents and clinical notes); conducted an inter-professional staff survey and used non-participant observation to analyse the utilisation and performance of hospital electronic systems and non-institutional resources to access and transfer key information.

## Results

Across the three general adult critical care units the mean time to access key patient data during the day-shift (outside rounding) were: laboratory values 27.4s (SD 20.2s); imaging archive 41s (SD 22.5s); PDF documents 26s (22.5s) and clinical notes (44.5s (SD 24.8s). Failure rates were 21.6%, 28.6%, 40.5% and 18.9% for these same electronic resources respectively. These speed testing data are shown in graph 1. Preliminary testing suggests that these indices of performance significantly deteriorate during the night shift and at weekends. During rounding, access times were further prolonged due to non-availability of work stations due to numbers of staff using electronic resources.

Barriers to using the hospital electronic systems were identified as lack of hardware; speed and access to software/institutional electronic resources; reliability of hardware (printers, mobile computer batteries, Wifi reliability, power socket availability); departmental use of paper-based ICU medical notes; and reliability of software (permissions/IT activation/update/corruption).

The inter-professional team used a mixture of *ad-hoc* (paper towels, scrap paper) and semi-formalised (posters, laminates, pre-printed proformas) paper resources in conjunction with personal hand-held devices to circumvent institutional system barriers. The internet was accessed via hand-held personal devices rather than trust systems in 88.2% of access attempts by doctors.

## Conclusions

Institutional electronic information resources performed slowly and unreliably with multiple barriers leading to use of informal *ad hoc* paper-based tools and personal hand-held devices. Taken together, this work identifies significant issues that will need to be addressed prior to implementation of a planned clinical information system for critical care.

**Figure 1:**
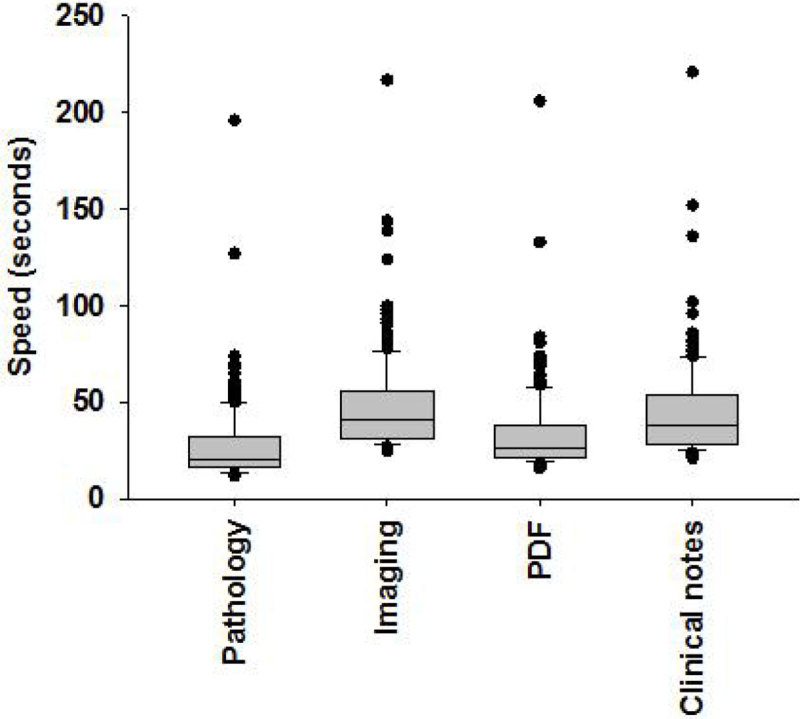
**Electronic resource access times**.
